# The establishment of the Household Air Pollution Consortium (HAPCO)

**DOI:** 10.3390/atmos10070422

**Published:** 2019-07-23

**Authors:** H. Dean Hosgood, Madelyn Klugman, Keitaro Matsuo, Alexandra J. White, Atsuko Sadakane, Xiao-Ou Shu, Ruy Lopez-Ridaura, Aesun Shin, Ichiro Tsuji, Reza Malekzadeh, Nolwenn Noisel, Parveen Bhatti, Gong Yang, Eiko Saito, Shafiur Rahman, Wei Hu, Bryan Bassig, George Downward, Roel Vermeulen, Xiaonan Xue, Thomas Rohan, Sarah K Abe, Philippe Broët, Eric J. Grant, Trevor J. B. Dummer, Nat Rothman, Manami Inoue, Martin Lajous, Keun-Young Yoo, Hidemi Ito, Dale P. Sandler, Habib Ashan, Wei Zheng, Paolo Boffetta, Qing Lan

**Affiliations:** 1 Department of Epidemiology and Population Health, Albert Einstein College of Medicine, Bronx, NY, 10461, United States; 2 Division of Epidemiology and Prevention, Aichi Cancer Center Research Institute; Nagoya, 464-8681, Japan; 3 Epidemiology Branch, National Institute of Environmental Health Science, Research Triangle Park, NC 27709, United States; 4 Department of Epidemiology, Radiation Effects Research Foundation, Hiroshima 732-0815, Japan; 5 Vanderbilt Institute for Global Health, Vanderbilt University School of Medicine, Nashville, TN 37203-1738, United States; 6 National Institute of Public Health, Cuernavaca, Morelos, 62100, Mexico; 7 Department of Preventative Medicine, Seoul National University College of Medicine, Seoul 03080, Korea; 8 Division of Epidemiology, Department of Health Informatics and Public Health, Tohoku University Graduate School of Medicine, Miyagi 980-8575, Japan; 9 Digestive Diseases Research Institute, Tehran University of Medical Sciences, Tehran, 14117, Iran; 10 CARTaGENE, Centre de Recherche du CHU Sainte-Justine, Montreal, Quebec, H3T 1C5, Canada; 11 BC Cancer, Vancouver, BC V5Z 1L3, Canada; 12 Center for Health Services, Vanderbilt University School of Medicine, Nashville, TN, 37203-1738, United States; 13 Division of Cancer Statistics and Integration, Center for Cancer Control and Information Services, National Cancer Center, Tokyo, 104-0045, Japan; 14 Department of Global Health Policy, Graduate School of Medicine, University of Tokyo, Tokyo, 113-8654, Japan; 15 Occupational and Environmental Epidemiology Branch, Division of Cancer Epidemiology and Genetics, National Cancer Institute, Bethesda MD 20892-7240; 16 Institute for Risk Assessment Services, Utrecht University, Utrecht, 3508, The Netherlands; 17 Epidemiology and Prevention Group, Center for Public Health Sciences, National Cancer Center, Tokyo, 104-0045, Japan; 18 School of Population and Public Health, University of British Columbia, Vancouver, BC, V6T 1Z3, Canada; 19 Department of Global Health and Population, Harvard T.H. Chan School of Public Health, Boston, MA; 20 Department of Health Sciences, The University of Chicago, Chicago, IL, 60637, United States; 21 The Tisch Cancer Institute, Mount Sinai School of Medicine, New York, NY 10029-6574, United States; 22 Department of Medical and Surgical Sciences, University of Bologna, Bologna, 40126, Italy

**Keywords:** pollution, environmental exposures, biomass, cohort studies, consortium, cancer

## Abstract

Household air pollution (HAP) is of public health concern with ~3 billion people worldwide (including >15 million in the US) exposed. HAP from coal use is a human lung carcinogen, yet the epidemiological evidence on carcinogenicity of HAP from biomass use, primarily wood, is not conclusive. To robustly assess biomass’s carcinogenic potential, prospective studies of individuals experiencing a variety of HAP exposures are needed. We have built a global consortium of 13 prospective cohorts (HAPCO: Household Air Pollution Consortium) that have site- and disease-specific mortality and solid fuel use data, for a combined sample size of 587,257 participants and 57,483 deaths. HAPCO provides a novel opportunity to assess the association of HAP with lung cancer death while controlling for important confounders such as tobacco and outdoor air pollution exposures. HAPCO is also uniquely positioned to determine the risks associated with cancers other than lung as well as non-malignant respiratory and cardiometabolic outcomes, for which prospective epidemiologic research is limited. HAPCO will facilitate research to address public health concerns associated with HAP-attributed exposures by enabling investigators to evaluate sex-specific and smoking status-specific effects under various exposure scenarios.

## 1. Introduction

Exposure to household air pollution (HAP), created by the combustion of coal, wood, and other forms of biomass for heating and cooking, is experienced by approximately 3 billion people worldwide [1] and causes over 3.5 million deaths per year [2,3]. In addition, one of its major components, polycyclic aromatic hydrocarbons (PAHs), is a known carcinogen [4]. Some of the strongest evidence on lung cancer caused by HAP is based on studies in Xuanwei, China. Xuanwei is ideal to study HAP-attributed lung cancer due to its high incidence rate of female lung cancer (~120 per 100,000 women)[5] combined with the fact that most women are never-smokers with substantial HAP exposures [6,7]. In Xuanwei, coal burning is associated with increased lung cancer risk [8] and improving stoves to mitigate HAP exposure is associated with significant reductions in lung cancer risk and lung cancer mortality [6,7]. A meta-analysis (>10,000 cases; >10,000 controls) of studies from Asia, Africa, Europe, and North America confirmed that significant increased risk of incident lung cancer was observed not only for coal burning in Southwestern China, which includes Xuanwei, but in areas throughout the world (OR=2.15; 95%CI=1.61–2.89; n=25), particularly in Asia (OR=2.27; 95%CI= 1.65–3.12; n=20) [9].

The International Agency for Research on Cancer (IARC) has classified HAP from indoor emissions from household coal combustion as a Group 1 human lung carcinogen [10]. However, due to limited epidemiologic evidence, IARC has classified HAP from biomass, primarily wood, as a probable human carcinogen (Group 2A) [11]. In an analysis pooling 7 case-control studies [12], it was suggested that individuals using wood to heat their homes experienced a higher risk of lung cancer than individuals who used non-solid fuels to heat their homes. With over 2.5 billion people exposed to wood smoke [1,10], and its increasing use for home heating in the US (over 15 million citizens currently exposed) [13], studies investigating the link between household wood smoke exposure and lung cancer are of tremendous importance.

Beyond the question of the pulmonary carcinogenicity of biomass, research priorities include elucidating the associations between HAP and cancer at other anatomical sites [14]. Retrospective studies have suggested that risk of cancers of the upper aero-digestive tract may be associated with exposures attributed to HAP. A review of 18 published studies evaluating the associations between HAP and cancers other than the lung found that HAP was associated with cervical, oral, nasopharyngeal, pharyngeal, and laryngeal cancers [15]. The observed associations between HAP and upper aero-digestive tract cancers remained significantly elevated when analyses were restricted to studies that controlled for smoking. In addition, a case-control study published subsequent to the meta-analysis found an association between HAP and breast cancer [16]. These associations, however, are considered preliminary as most studies had limited sample size, and all were retrospective in design. Moreover, additional research is needed to robustly assess the non-malignant health outcomes that have been reported in the literature to be associated with the use of solid fuel for heating and cooking in the home. Several systematic reviews have assessed the association between HAP exposures and chronic diseases other than cancer, including respiratory and cardiovascular diseases[17–21]. Similar to systematic reviews of the existing cancer literature, these reviews observed inconclusive findings and that the literature is limited in scope with regards to prospective studies, as well as a variety of exposure scenarios.

There is a need for large-scale prospective data that can assess the relationship between HAP and health outcomes, while adjusting for important demographic and clinical factors. However, most prospective studies are limited in the number of ascertained disease outcomes. A consortium effort that brings together multiple prospective studies may lead to a sufficient number of cases of malignant and non-malignant diseases to assess many of the previously mentioned HAP-disease relationships that have been inconclusive in the literature to date.

## 2. Materials and Methods

Prospective longitudinal cohort studies provide an excellent opportunity to assess the impact of HAP on chronic disease. To accurately quantify the relative and absolute risks of carcinogenic and cardiometabolic diseases associated with HAP exposure, particularly biomass-derived, we have established the Household Air Pollution Consortium (HAPCO). To date, 13 cohorts have agreed to participate (Table 1). These cohorts include: Three Prefecture Cohort Study Aichi (Aichi) [22], British Columbia Generations Project (BCGP) [23], CARTaGENE (CaG) [24], Golestan Cohort (GCS) [25], Health Effects of Arsenic Longitudinal Study (HEALS) [26], Korea Multi-center Cancer Cohort (KMCC) [27], Three Prefecture Cohort Study Miyagi (Miyagi) [22], Mexican Teachers Cohort (MTC) [28], RERF life-span study (RERF) [29], Sister Study (SIS) [30], Shanghai Men’s Health Study (SMHS) [31], Shanghai Women’s Health Study (SWHS) [32], and Xuanwei Cohort Study (XW) [6]. Almost all cohorts have accrued more than 5 years of follow-up data (with many having accrued 10–20 years) and all cohorts have more than 15,000 participants. In all cohorts, participants filled out baseline questionnaires that collected information about, among many other things, their fuel use for heating and/or cooking.

Given that no direct HAP exposure air monitoring measurements are available beyond the questionnaires, the baseline questionnaires were used to initially categorize individuals from each cohort into ever/never solid fuel users for heating or cooking in the home. Eight studies directly asked each participant if they have ever used solid fuels in their homes for heating or cooking. This variable was used to classify participants as ever/never users in these studies. In the remaining studies, individuals with high exposure levels were classified as ever exposed and individuals with low exposure levels were classified as never exposed. For 2 studies, those with any years of coal or wood use were classified as ever users, while those with zero years of use were classified as never users. For 3 studies, the ever users were those who used solid fuel in a stove without a chimney, and the never users were those who used a stove with a chimney. As described below, future aims of HAPCO include refining these harmonized exposure variables, and possibly correlating questionnaire data with quantified biomarkers of HAP exposure (i.e., urinary PAH levels).

## 3. Results

In total, there are data from 587,257 participants (348,146 participants in cohorts conducted in Asia; 239,111 participants in cohorts conducted in North America), of whom 73.5% are females (220,903 females in cohorts conducted in Asia; 210,472 females in cohorts conducted in North America) and 62.0% are never-smokers (214,520 never-smokers in cohorts conducted in Asia; 149,610 never-smokers in cohorts conducted in North America) (Table 1). Overall, the mean age at baseline among all cohorts tended to be in the mid-50’s, except for HEALS, XW, and MTC, which were slightly younger. The average BMI at baseline tended to be lower among the cohorts conducted in Asia compared to those conducted in North America. Given the maturity of many of these cohorts, there has been a large number of deaths (n = 57,483). To date, there have been a total of 8,347 lung cancer deaths, as well as 306 oral and nasopharyngeal, 852 esophageal, and 154 laryngeal cancer deaths (Table 2). There have also been a total of 25,465 cardiovascular deaths.

About a third of all subjects experienced HAP attributed to solid fuel use (Table 3). The prevalence of biomass (wood) use was 18.6% in all cohorts (25.3% in cohorts conducted in Asia, 8.8% in cohorts conducted in North America) and coal use was 13.4% (26.2% in cohorts conducted in Asia, 2.1% in cohorts conducted in North America). Regarding additional data available for harmonization, the number of years of use for each fuel type is available for 73% of subjects, and data related to the presence of ventilation in the vicinity of the fuel use and the type of stove used are available for 70% of subjects (Table 4).

The original, baseline questionnaire data from the cohorts will be harmonized to allowing for pooling the HAP exposures in terms of the intensity and duration of exposure. Each HAPCO cohort has the covariate data for our analyses (Table 4), such as age, gender, active tobacco smoking, education, and BMI. These variables will be harmonized to ensure comparability across cohorts. For example, active tobacco smoking will be first classified granularly (ever, never), where never smoking is defined has not having smoked more than 100 cigarettes in their lifetime. Smoking will also be assessed by years of smoking and pack-years smoked. Finally, years since quitting smoking will also be harmonized, which will also allow for classification of individuals as current, former, and never smokers. In addition to these covariates, OAP exposures will be estimated by GIS-based methods that link geocoded residential addresses to exposure databases. Air pollution estimates will be available for all cohorts either through local models and/or via the global air pollution maps for PM_2.5_ and NO_2_. The CaG and BCGP cohorts are covered by air pollution exposure surfaces as generated and collated within the CANUE project[33]. The SIS cohort has already estimated PM_2.5_ and NO_2_ concentration through national, satellite-based models[34]. PM_2.5_ and NO_2_ concentrations for the other cohorts are currently being derived using satellite-based estimates as well as from available ground-level monitoring data. Once data harmonization is complete, the mortality rates of each disease outcome of interest will be assessed overall, by sex, and by smoking status. Next, the associations between HAP exposure and mortality from lung, esophageal, oral, pharyngeal, and laryngeal cancers, by smoking status and sex, will be assessed.

## 4. Conclusions

HAPCO currently involves 13 cohorts from 8 countries on 2 continents. Major strengths of HAPCO include the use of prospectively designed studies that substantially mitigate recall and temporal biases, as well as the large sample size lending itself to high statistical power. A collaborative data harmonization procedure for exposures, potential confounders, and outcomes will be developed for accurate estimation of magnitude and type of HAP exposure. By providing this platform, HAPCO will facilitate research by investigators to address public health concerns associated with HAP-attributed exposures. A robust assessment of exposures may serve to better predict risk of cancer and/or cardiovascular disease, which may impact future local, region, and national policies. Future aims of HAPCO are to (1) identify additional cohort studies to join the consortium, particularly cohorts conducted in Africa and South America where solid fuel use remains prevalent, and (2) quantitatively assess potential biomarkers for HAP exposure. HAPCO also welcomes additional project proposals from interested researchers. Such proposals will be reviewed by, and individually approved by, each cohort before access to the respective cohort data can be facilitated by HAPCO. These studies will have the potential to inform future interventions to reduce disease burden in global populations.

## Figures and Tables

**Table 1.** Characteristics of Cohorts Participating in HAPCO (Household Air Pollution Consortium).

Cohort (Abbreviation)	Country	Enrollment Dates	Total Subject	Males		Females		Never Smokers		Ever Smokers		Age at Baseline		BMI at Baseline (kg/m^2^)	

				N	%	N	%	N	%	N	%	Mean	Range	Mean	Range
**Cohorts conducted in Asia**															
Golestan Cohort (GCS)	Iran	2004–2008	50045	21221	42.4%	28824	57.6%	39035	78.0%	11010	22.0%	52.1	40.0–75.0	26.7	11.8–62.2
Health Effects of Arsenic Longitudinal Study (HEALS)	Bangladesh	2000–2002	16386	0	0.0%	16386	100.0%	13928	85.0%	2458	15.0%	37.1	17.0–75.0	19.8	9.7–51.8
Korea Multi-center Cancer Cohort (KMCC)	Korea	1993–2004	20636	8235	39.9%	12401	60.1%	12819	62.1%	7471	36.2%	54.1	15.0–99.0	23.1	9.7–51.8
RERF life-span study (RERF)	Japan	1963–1993	52883	20390	38.6%	32493	61.4%	28408	53.7%	22102	41.8%	52.2	19.3–98.7	21.7	10.4–74.0
Shanghai Men’s Health Study (SMHS)	China	2002–2006	25958	25958	100.0%	0	0.0%	9046	34.8%	16912	65.2%	59.3	40.1–75.0	23.9	11.6–40.3
Shangai Women’s Health Study (SWHS)	China	1996–2000	74942	0	0.0%	74942	100.0%	72829	97.2%	2113	2.8%	52.6	40.0–71.0	24.0	12.7–49.0
Three Prefecture Cohort Study Aichi (Aichi)	Japan	1985	33529	15746	47.0%	17783	53.0%	15285	45.6%	15723	46.9%	56.4	40.0–103.0	22.1	10.6–57.0
Three Prefecture Cohort Study Miyagi (Miyagi)	Japan	1984	31345	13992	44.6%	17353	55.4%	12704	40.5%	18641	59.5%	57.3	40.0–98.0	23.2	7.2–64.9
Xuanwei Cohort Study (XW)	China	1992–1996	42422	21701	512%	20721	48.8%	22351	52.7%	20071	47.3%	39.5	25.0–59.0	not available	
**Sub-Totals for Cohorts in Asia**			**348146**	**127243**	**36.5%**	**220903**	**63.5%**	**214520**	**61.6%**	**97773**	**28.1%**				
**Cohorts conducted in North America**															
British Columbia Generations Project (BCGP)	Canada	2008–2016	29852	9345	313%	20462	68.5%	15321	51.3%	13412	44.9%	56.4	31.0–74.0	26.4	10.3–62.1
CARTaGENE (CaG)	Canada	2009–2016	43061	19249	44.7%	23812	55.3%	17259	40.1%	23434	54.4%	53.6	38.3–73.1	27.6	11.3–125.1
Mexican Teachers Cohort (MTC)	Mexico	2006–2008	115314	0	0.0%	115314	100.0%	90061	78.1%	25254	21.9%	43.0	20.0–84.0	27.4	10.2–57.8
Sister Study (SIS)	United States	2003–2009	50884	0	0.0%	50884	100.0%	26969	53.0%	23915	47.0%	55.6	35.0–76.5	27.8	11.5–72.1
**Sub-Totals for Cohorts in North America**			**239111**	**28594**	**12.0%**	**210472**	**88.0%**	**149610**	**62.6%**	**86015**	**36.0%**				
**Totals for all Cohort Contributing Data**			**587257**	**155837**	**26.5%**	**431375**	**73.5%**	**364130**	**62.0%**	**183788**	**31.3%**				

**Table 2.** Number of selected cancer-related deaths in HAPCO (Household Air Pollution Consortium).

	Lung			Oral and nasopharyngeal	Esophageal	Laryngeal
	
	Men	Women	All	All	All	All
**Cohorts conducted in Asia**	4694	3478	8172	293	832	147
**Cohorts conducted in North America**	113	62	175	13	20	7

**All Cohorts**	**4807**	**3540**	**8347**	**306**	**852**	**154**

**Table 3.** Prevalence of household air pollution exposures in HAPCO (Household Air Pollution Consortium).

	All subjects	Clean Fuel† Users		Coal Users		Biomass¥ Users		Solid Fuel¶ Users	
	
	n	n	%	n	%	n	%	n	%
**Cohorts conducted in Asia**	348146	162963	46.8%	91067	26.2%	88134	25.3%	178965	51.4%
**Cohorts conducted in North America**	239111	226870	94.9%	4942	2.1%	21045	8.8%	25987	10.9%

**All Cohorts**	**587257**	**381129**	**64.9%**	**78755**	**13.4%**	**109179**	**18.6%**	**187698**	**32.0%**

† includes all non-solid fuels (i.e., electric, gas)

¥ includes wood and other biomass sources such as dung cakes and charcoal briquettes

¶ includes coal and biomass users, which are not mutally exclusive (i.e., some individuals use both coal and biomass)

**Table 4.** Data available in each HAPCO (Household Air Pollution Consortium) cohort.

	HEALS	SWHS	GCS	KMCC	Aichi	Miyagi	RERF	XW	CaG	BCGP	MTC	SIS	SMHS
Age at Baseline (years)	√	√	√	√	√	√	√	√	√	√	√	√	√
BMI at Baseline (kg/m2)	√	√	√	√	√	√	√	√	√	√	√	√	√
Educational Attainment	√	√	√	√	√	√	√	√	√	√	√	√	√
Tobacco Smoking Status (ever/never)	√	√	√	√	√	√	√	√	√	√	√	√	√
Pack-years smoked	√	√	√	√	√	√	√	√	√	√	√	√	√
Solid Fuel Use	√	√	√	√	√	√	√	√	√	√	√	√	√
Fuel Type	√	√	√	√	√	√	√	√	√	√	√	√	√
Stove Type	√	√	√	√	√	√		√	√	√		√	√
Use of Ventilation	√	√	√		√	√		√	√	√		√	√
Years of HAP Exposure	√	√	√				√	√			√	√	√
OAP (NO2, PM2.5)	¥	¥	¥	¥	¥	¥	¥	¥	√	√	¥	√	¥

¥ Currently being derived

## References

[1] Ezzati M; Organization., W.H. Comparative quantification of health risks : global and regional burden of disease attributable to selected major risk factors; World Health Organization: Geneva, 2004.

[2] Smith KR; Bruce N; Balakrishnan K; Adair-Rohani H; Balmes J; Chafe Z; Dherani M; Hosgood HD; Mehta S; Pope D, Millions dead: how do we know and what does it mean? Methods used in the comparative risk assessment of household air pollution. Annual Review: Public Health 2014, 35, 185–206, doi:10.1146/annurev-publhealth-032013-182356. 24641558

[3] Global, regional, and national comparative risk assessment of 84 behavioural, environmental and occupational, and metabolic risks or clusters of risks, 1990–2016: a systematic analysis for the Global Burden of Disease Study 2016. Lancet 2017, 390, 1345–1422, doi:10.1016/s0140-6736(17)32366-8. 28919119 PMC5614451

[4] IARC Working Group on the Evaluation of Carcinogenic Risk to Humans: Some Non-heterocyclic Polycyclic Aromatic Hydrocarbons and Some Related Exposures In IARC Monographs on the Evaluation of Carcinogenic Risks to Humans, International Agency for Research on Cancer: Lyon, France, 2010; Vol. 92. PMC4781319 21141735

[5] Xiao Y; Shao Y; Yu X; Zhou G The epidemic status and risk factors of lung cancer in Xuanwei City, Yunnan Province, China. Frontiers of medicine 2012, 6, 388–394, doi:10.1007/s11684-012-0233-3. 23224416

[6] Lan Q; Chapman RS; Schreinemachers DM; Tian L; He X Household stove improvement and risk of lung cancer in Xuanwei, China. J Natl Cancer Inst 2002, 94, 826–835. 12048270 10.1093/jnci/94.11.826

[7] Hosgood HD; Chapman R; Shen M; Blair A; Chen E; Zheng T; Lee KM; He X; Lan Q Portable stove use is associated with lower lung cancer mortality risk in lifetime smoky coal users. Brit J Cancer 2008, 99, 1934–1939, doi:DOI 10.1038/sj.bjc.6604744. 19034286 PMC2600700

[8] Lan Q; He X; Shen M; Tian L; Liu LZ; Lai H; Chen W; Berndt SI; Hosgood HD; Lee KM, Variation in lung cancer risk by smoky coal subtype in Xuanwei, China. Int J Cancer 2008, 123, 2164–2169, doi:10.1002/ijc.23748. 18712724 PMC2974309

[9] Hosgood HD 3rd; Wei H; Sapkota A; Choudhury I; Bruce N; Smith KR; Rothman N; Lan Q Household coal use and lung cancer: systematic review and meta-analysis of case-control studies, with an emphasis on geographic variation. International Journal of Epidemiology 2011, 40, 719–728, doi:10.1093/ije/dyq259. 21278196 PMC3147068

[10] IARC. Household Use of Solid Fuels and High-temperature Frying; World Health Organization: Lyon, 2010; Vol. 95. PMC5046077 20701241

[11] Straif K; Baan R; Grosse Y; Secretan B; El Ghissassi F; Cogliano V; Group WHOIA f.R.o.C.M.W. Carcinogenicity of household solid fuel combustion and of high-temperature frying. Lancet Oncol 2006, 7, 977–978. 17348122 10.1016/s1470-2045(06)70969-x

[12] Hosgood HD 3rd; Boffetta P; Greenland S; Lee YC; McLaughlin J; Seow A; Duell EJ; Andrew AS; Zaridze D; Szeszenia-Dabrowska N, In-home coal and wood use and lung cancer risk: a pooled analysis of the International Lung Cancer Consortium. Environmental Health Perspective 2010, 118, 1743–1747, doi:10.1289/ehp.1002217. PMC3002194 20846923

[13] Rogalsky DK; Mendola P; Metts TA; Martin WJ 2nd., Estimating the number of low-income americans exposed to household air pollution from burning solid fuels. Environ Health Perspect 2014, 122, 806–810, doi:10.1289/ehp.1306709. 24833615 PMC4123020

[14] Reid BC; Ghazarian AA; DeMarini DM; Sapkota A; Jack D; Lan Q; Winn DM; Birnbaum LS Research opportunities for cancer associated with indoor air pollution from solid-fuel combustion. Environ Health Perspect 2012, 120, 1495–1498, doi:10.1289/ehp.1204962. 22846419 PMC3556624

[15] Josyula S; Lin J; Xue X; Rothman N; Lan Q; Rohan TE; Hosgood HD 3rd. Household air pollution and cancers other than lung: a meta-analysis. Environmental Health 2015, 14, 24, doi:10.1186/s12940-015-0001-3. 25890249 PMC4377187

[16] White AJ; Teitelbaum SL; Stellman SD; Beyea J; Steck SE; Mordukhovich I; McCarty KM; Ahn J; Rossner P Jr.; Santella RM, Indoor air pollution exposure from use of indoor stoves and fireplaces in association with breast cancer: a case-control study. Environmental health : a global access science source 2014, 13, 108, doi:10.1186/1476-069x-13-108. 25495350 PMC4320487

[17] Assad NA; Balmes J; Mehta S; Cheema U; Sood A Chronic obstructive pulmonary disease secondary to household air pollution. Semin Respir Crit Care Med 2015, 36, 408–421, doi:10.1055/s-0035-1554846. 26024348

[18] Kurmi OP; Sadhra CS; Ayres JG; Sadhra SS Tuberculosis risk from exposure to solid fuel smoke: a systematic review and meta-analysis. J Epidemiol Community Health 2014, 68, 1112–1118, doi:10.1136/jech-2014-204120. 25081627

[19] Jary H; Simpson H; Havens D; Manda G; Pope D; Bruce N; Mortimer K Household Air Pollution and Acute Lower Respiratory Infections in Adults: A Systematic Review. PLoS One 2016, 11, e0167656, doi:10.1371/journal.pone.0167656. 27907205 PMC5132292

[20] Fatmi Z; Coggon D Coronary heart disease and household air pollution from use of solid fuel: a systematic review. Br Med Bull 2016, 118, 91–109, doi:10.1093/bmb/ldw015. 27151956 PMC4973663

[21] West SK; Bates MN; Lee JS; Schaumberg DA; Lee DJ; Adair-Rohani H; Chen DF; Araj H Is household air pollution a risk factor for eye disease? Int J Environ Res Public Health 2013, 10, 5378–5398, doi:10.3390/ijerph10115378. 24284355 PMC3863851

[22] Marugame T; Sobue T; Satoh H; Komatsu S; Nishino Y; Nakatsuka H; Nakayama T; Suzuki T; Takezaki T; Tajima K, Lung cancer death rates by smoking status: comparison of the Three-Prefecture Cohort study in Japan to the Cancer Prevention Study II in the USA. Cancer science 2005, 96, 120–126, doi:10.1111/j.1349-7006.2005.00013.x. 15723657 PMC11158599

[23] Dhalla A; McDonald TE; Gallagher RP; Spinelli JJ; Brooks-Wilson AR; Lee TK; Lai C; Borugian MJ; Woods RR; Le ND, Cohort Profile: The British Columbia Generations Project (BCGP). Int J Epidemiol 2018, 10.1093/ije/dyy160, doi:10.1093/ije/dyy160. 30169793

[24] Awadalla P; Boileau C; Payette Y; Idaghdour Y; Goulet JP; Knoppers B; Hamet P; Laberge C Cohort profile of the CARTaGENE study: Quebec’s population-based biobank for public health and personalized genomics. Int J Epidemiol 2013, 42, 1285–1299, doi:10.1093/ije/dys160. 23071140

[25] Pourshams A; Khademi H; Malekshah AF; Islami F; Nouraei M; Sadjadi AR; Jafari E; Rakhshani N; Salahi R; Semnani S, Cohort Profile: The Golestan Cohort Study--a prospective study of oesophageal cancer in northern Iran. Int J Epidemiol 2010, 39, 52–59, doi:10.1093/ije/dyp161. 19332502 PMC3709199

[26] Ahsan H; Chen Y; Parvez F; Argos M; Hussain AI; Momotaj H; Levy D; van Geen A; Howe G; Graziano J Health Effects of Arsenic Longitudinal Study (HEALS): description of a multidisciplinary epidemiologic investigation. Journal of exposure science & environmental epidemiology 2006, 16, 191–205, doi:10.1038/sj.jea.7500449. 16160703

[27] Yoo KY; Shin HR; Chang SH; Lee KS; Park SK; Kang D; Lee DH Korean Multi-center Cancer Cohort Study including a Biological Materials Bank (KMCC-I). Asian Pac J Cancer Prev 2002, 3, 85–92. 12718614

[28] Lajous M; Ortiz-Panozo E; Monge A; Santoyo-Vistrain R; Garcia-Anaya A; Yunes-Diaz E; Rice MS; Blanco M; Hernandez-Avila M; Willett WC, Cohort Profile: The Mexican Teachers’ Cohort (MTC). Int J Epidemiol 2015, 10.1093/ije/dyv123, doi:10.1093/ije/dyv123. 26337903

[29] Ozasa K; Shimizu Y; Suyama A; Kasagi F; Soda M; Grant EJ; Sakata R; Sugiyama H; Kodama K Studies of the mortality of atomic bomb survivors, Report 14, 1950–2003: an overview of cancer and noncancer diseases. Radiation research 2012, 177, 229–243. 22171960 10.1667/rr2629.1

[30] Sandler DP; Hodgson ME; Deming-Halverson SL; Juras PS; D’Aloisio AA; Suarez LM; Kleeberger CA; Shore DL; DeRoo LA; Taylor JA, The Sister Study Cohort: Baseline Methods and Participant Characteristics. Environ Health Perspect 2017, 125, 127003, doi:10.1289/EHP1923. 29373861 PMC5963586

[31] Shu XO; Li H; Yang G; Gao J; Cai H; Takata Y; Zheng W; Xiang YB Cohort Profile: The Shanghai Men’s Health Study. International journal of epidemiology 2015, 44, 810–818, doi:10.1093/ije/dyv013. 25733578 PMC4521127

[32] Zheng W; Chow WH; Yang G; Jin F; Rothman N; Blair A; Li HL; Wen W; Ji BT; Li Q, The Shanghai Women’s Health Study: rationale, study design, and baseline characteristics. Am J Epidemiol 2005, 162, 1123–1131, doi:10.1093/aje/kwi322. 16236996

[33] Hystad P; Demers PA; Johnson KC; Brook J; van Donkelaar A; Lamsal L; Martin R; Brauer M Spatiotemporal air pollution exposure assessment for a Canadian population-based lung cancer case-control study. Environmental Health 2012, 11, 22, doi:10.1186/1476-069x-11-22. 22475580 PMC3372423

[34] Chan SH; Van Hee VC; Bergen S; Szpiro AA; DeRoo LA; London SJ; Marshall JD; Kaufman JD; Sandler DP Long-Term Air Pollution Exposure and Blood Pressure in the Sister Study. Environ Health Perspect 2015, 123, 951–958, doi:10.1289/ehp.1408125. 25748169 PMC4590742

[35] Larkin A; Geddes JA; Martin RV; Xiao Q; Liu Y; Marshall JD; Brauer M; Hystad P Global Land Use Regression Model for Nitrogen Dioxide Air Pollution. Environ Sci Technol 2017, 51, 6957–6964, doi:10.1021/acs.est.7b01148. 28520422 PMC5565206

